# Cholangiolocarcinoma with Ductal Plate Malformation Pattern: A 6-Year Follow-Up

**DOI:** 10.70352/scrj.cr.25-0086

**Published:** 2025-05-01

**Authors:** Yuta Ushida, Gen Sugawara, Takayuki Minami, Yoriko Yamashita, Masaya Inoue

**Affiliations:** 1 Department of Surgery, Toyota Kosei Hospital, Toyota, Aichi, Japan; 2 Department of Pathology, Toyota Kosei Hospital, Toyota, Aichi, Japan

**Keywords:** cholangiolocarcinoma, ductal plate malformation, hepatocellular carcinoma

## Abstract

**INTRODUCTION:**

Cholangiolocarcinoma (CLC) with ductal plate malformation (DPM) is a rare primary liver cancer originating from the canals of Hering. It often exhibits intermediate behavior between hepatocellular carcinoma and intrahepatic cholangiocarcinoma. Diagnosing CLC with DPM is challenging due to overlapping imaging features with other liver malignancies.

**CASE PRESENTATION:**

An 82-year-old man under surveillance for bladder cancer was incidentally found to have a liver nodule in segment 8. Over 6 years, the lesion grew from 10 mm to 41 mm and showed dynamic changes on imaging. Despite two inconclusive biopsies, a diagnosis of CLC with DPM was confirmed after a third biopsy and consultation with a specialized institution. The patient underwent a right hepatectomy, and pathological examination confirmed CLC with DPM. No evidence of recurrence was observed 19 months post-surgery.

**CONCLUSIONS:**

This case underscores the importance of long-term follow-up and a multidisciplinary approach in managing rare hepatic malignancies. The clinical course provides valuable insights into the progression of CLC with DPM and may aid in diagnosing similar challenging cases.

## Abbreviations

ARID1A
: AT-rich interactive domain-containing protein 1A
CLC
: cholangiolocarcinoma
CT
: computed tomography
DPM
: ductal plate malformation
FGFR2
: fibroblast growth factor receptor 2
HCC
: hepatocellular carcinoma
ICC
: intrahepatic cholangiocarcinoma
MRI
: magnetic resonance imaging
US
: ultrasound

## INTRODUCTION

Cholangiolocarcinoma (CLC) with a ductal plate malformation (DPM) pattern is a rare and distinct subtype of primary liver carcinoma, characterized by irregular and tortuous glandular structures resembling biliary lesions seen in Caroli disease.^1,2)^ Diagnosing CLC is challenging due to its nonspecific imaging features, which often result in misdiagnosis as hepatocellular carcinoma.^3)^ This report presents a rare case of CLC with a DPM pattern that was monitored over 6 years through comprehensive imaging and pathological evaluations.

## CASE PRESENTATION

**Fig. 1:**
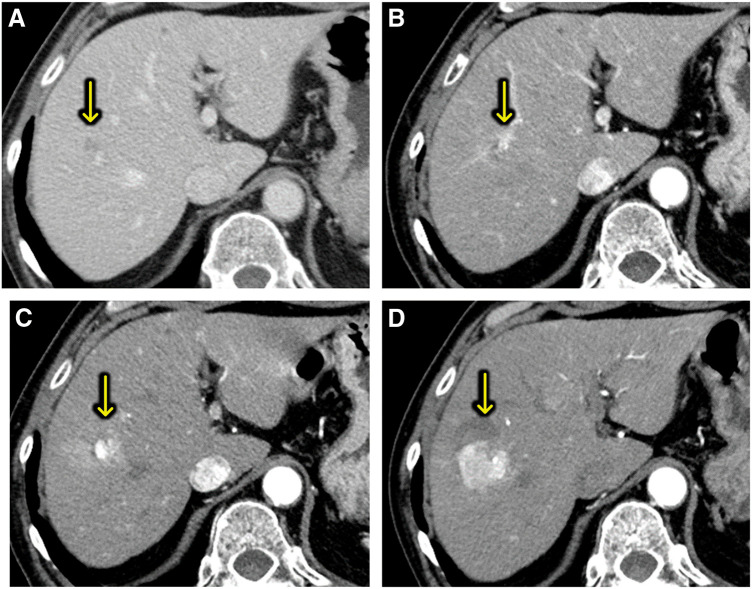
Serial computed tomography (CT) imaging findings of liver tumor. (**A**) Initial CT imaging revealed a low-attenuation nodule in segment 8. (**B**) After 2.5 years, the lesion showed early enhancement and measured 14.2 mm, with a doubling time of 436 days. (**C**) At 4 years, the tumor further increased in size to 17.6 mm and demonstrated early arterial enhancement and washout. (**D**) By the sixth year, the tumor had grown to 40.6 mm, with heterogeneous enhancement and evidence of vascular penetration through the tumor.

**Fig. 2:**
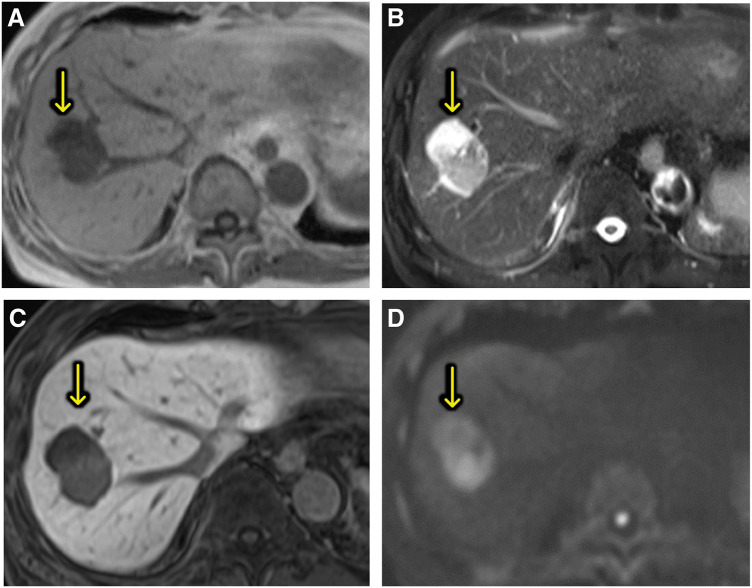
Magnetic resonance imaging findings of liver tumor. (**A**) T1-weighted imaging revealed a hypointense tumor (**B**) T2-weighted imaging showed relatively high signal intensity. (**C**) In the hepatobiliary phase, the tumor exhibited hypointensity. (**D**) Diffusion-weighted imaging demonstrated hyperintensity of the tumor.

**Fig. 3:**
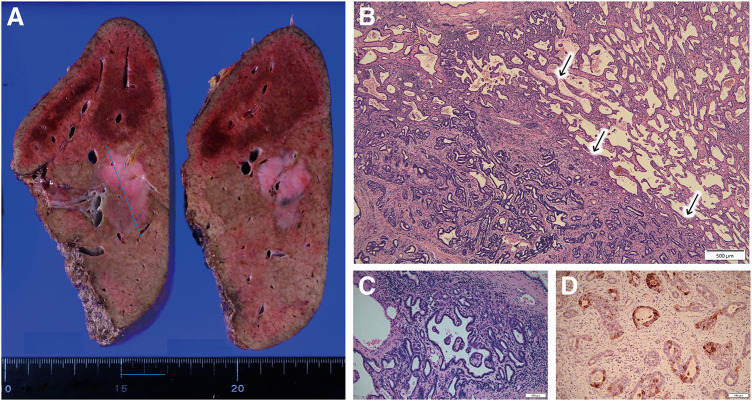
Macroscopic and microscopic findings of the resected tumor. (**A**) Grossly, the tumor was a well-circumscribed lesion located in segment 8, measuring 42 mm. (**B**) Microscopically, two components were identified: a small duct-type Intrahepatic cholangiocarcinoma and a benign-looking lesion. (**C**) A ductal plate malformation pattern was observed in the benign-looking area, supporting the transformation hypothesis. (**D**) Neural cell adhesion molecule (NCAM) staining was positive, consistent with the characteristics of cholangiolocarcinoma.

**Fig. 4:**
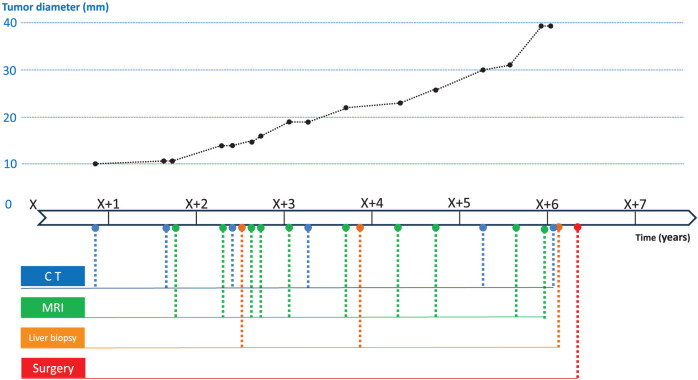
Clinical course of the liver tumor. Timeline illustrating tumor progression over several years. The *x*-axis represents time (years), whereas the *y*-axis indicates tumor diameter (mm). Key clinical interventions, including computed tomography (CT), magnetic resonance imaging (MRI), liver biopsy, and surgery, were marked along the timeline. The tumor showed gradual enlargement with significant growth observed over time.

An 82-year-old man with a history of hypertension, colon cancer, bladder cancer, and childhood asthma underwent a routine postoperative follow-up for bladder cancer. He was asymptomatic at the time, but a computed tomography (CT) scan revealed an 8 × 10 mm low-attenuation nodule in segment 8, initially suspected to be a hemangioma (**Fig. 1A**). Approximately 2.5 years later, the lesion increased to 14 mm and showed early enhancement with portal washout on dynamic CT (**Fig. 1B**). A liver biopsy stage revealed hepatic tissue with partial fibrous but minimal tumor-related changes and no clear malignancy. Although the enlarging tumor raised suspicion of malignancy, the patient and family opted for observation until a definitive diagnosis was made, given the patient’s age and general health. Tumor markers were monitored over time, but no abnormal values were observed. Another biopsy conducted approximately 4 years after the initial detection, prompted by further enlargement to 18 mm, revealed a tumor with early enhancement and washout, similar to hepatocellular carcinoma (HCC) (**Fig. 1C**). However, no clear evidence of malignancy was confirmed, though biliary hamartoma was suspected. Histological analysis showed scattered bile ducts lined by a single-layered epithelium. Immunohistochemical was negative for p53, CD31, and CD34 with an MIB-1 labeling index of 3%–5%. Although the lesion displayed minimal atypia, its size (20 mm) was atypical for a typical hamartoma. Six years after initial detection, the lesion had grown to 41 mm and exhibited heterogeneous enhancement, vascular penetration, and no peripheral bile duct dilation, distinguishing it from ordinary intrahepatic cholangiocarcinoma (ICC) and HCC (**Fig. 1D**). Magnetic resonance imaging (MRI) showed hypointensity on T1-weighted imaging (**Fig. 2A**) and relatively high intensity on T2-weighted imaging (**Fig. 2B**). It also exhibited hypointensity in the hepatobiliary phase (**Fig. 2C**) and hyperintensity in diffusion-weighted imaging (**Fig. 2D**). These led to a third biopsy and consultation with a specialized institution. The tumor showed neoplastic proliferation of the small bile ducts with moderate fibrous stroma. The biliary epithelium was cuboidal with hyperchromatic nuclei showing mild anisokaryosis. Immunohistochemically was negative for MUC6 and dPAS consistent with well-differentiated CLCs. Retrospective analysis suggested that the second biopsy had sampled a benign-appearing part of the CLC rather than a separate biliary hamartoma. Following the diagnosis of CLC, the patient consented to surgical treatment. The tumor was located at the root of the anterior and posterior Glisson pedicles, and a right hepatic resection was selected. Lymph node dissection was performed according to the standard oncologic procedure and the extrahepatic bile duct was preserved. Liver function tests (indocyanine green retention rate at 15 minutes: 9%; indocyanine green disappearance rate: 0.168) and volumetric analysis (remnant liver volume: 392 mL, 38.5%) indicated that surgery could be safely tolerated. Surgery lasted 217 minutes, with a blood loss of 219 mL and no transfusion required. Postoperatively, he developed a Grade B bile leak, which was managed conservatively. He was discharged on postoperative day 29. Macroscopically, the resected specimen exhibited a well-circumscribed solid tumor in segment 8. The tumor measured 42 × 20 mm with a central whitish to pink area surrounded by fibrotic tissue (**Fig. 3A**). Microscopically, the tumor consisted of a small duct-type ICC and a benign-looking lesion (**Fig. 3B**). It is composed of proliferating small-to medium-sized tumor glands with mild-to-moderate atypia, and the stroma consists of fibrous tissue. The peripheral region exhibited features of ductal plate malformation (**Fig. 3C**) characterized by irregularly shaped biliary epithelial lesions with minimal atypia and preserved portal tracts. These findings suggest a transformation from a ductal plate malformation pattern to conventional small duct-type ICC. Immunohistochemical staining was positive for Neural Cell Adhesion Molecule (**Fig. 3D**). The final diagnosis was CLC, classified as pT2N0M0 or pStage II according to the Union for International Cancer Control 8th edition. The clinical course of this case is illustrated in **Fig. 4**. Serial CT and MRI examinations were performed at regular intervals, and three biopsies were conducted at key time points, ultimately leading to a definitive diagnosis and surgical intervention. At the 19-month postoperative follow-up, no recurrence was observed.

## DISCUSSION

This case highlights three notable features. First, we observed the progression of this rare tumor through imaging over 6 years. The lesion initially appeared as a cystic tumor but later developed enhanced nodules and vascular penetration through the tumor, demonstrating the dynamic nature of CLC. Second, the challenges of biopsy were evident requiring three attempts. The second biopsy likely sampled a benign-looking area of the CLC, delaying a definitive diagnosis. Third, surgical treatment achieved favorable outcomes, with no recurrence observed to date. This case provides a rare opportunity to observe the natural history of CLC over time, from initial detection to definitive diagnosis and treatment.

CLC is a rare type of liver cancer, accounting for 0.56% of all resected primary liver cancers.^4)^ According to the WHO Classification of Tumors of the Digestive System, 5th Edition, small duct-type cholangiocarcinoma is a distinct subtype of intrahepatic cholangiocarcinoma.^5)^ First described by Steiner and Higginson in 1959, CLCs originate from the cholangioles or canals of Hering.^6)^ Advances in hepatic progenitor cell research have provided evidence that CLCs originate from these cells. Nakamura et al. proposed that ICC with a predominant DPM has a distinct pattern from the conventional ICC and is classified as a subtype of CLC.^1)^ In this case, consultation with this institution confirmed the final diagnosis of CLC with DPM.

Diagnosing CLC is challenging due to its overlapping imaging features with other hepatic malignancies, particularly HCC.^3)^ On ultrasound (US), it typically appears as a heterogeneous hypoechoic lesion with an unclear border.^7)^ Contrast-enhanced US with Sonazoid often shows hypervascular enhancement from the interior or periphery of the entire tumor without rapid washout in the arterial-portal dominant phase and a distinct post-vascular defect.^8)^ MRI shows low T1, high T2, and high diffusion-weighted signal intensities. On dynamic CT or MRI, CLCs generally exhibit early-phase peripheral or global hyperenhancement and late-phase hyperenhancement.^9,10)^ Consistent observations across these modalities is portal venules penetrating the lesion due to tumor cell replacement by normal liver cells, with no peripheral bile duct dilatation.^10,11)^

Imaging may suggest irregular glandular structures; however, histopathology remains the gold standard for diagnosis. Histologically, CLCs exhibit a ductular reaction-like appearance, characterized by anastomosing small glands embedded within an abundant fibrous stroma.^12)^ The tumor often follows a stepwise progression transitioning from benign DPM to biliary intraepithelial neoplasia, ultimately leading to malignant transformation to CLC.^13)^ CLCs typically present as small well-defined lesions that frequently demonstrate cystic changes and pushing growth pattern.^13)^ It is characterized by low-grade cytology, a low Ki-67 proliferation index, and histopathological features^13,14)^ that distinguish it from conventional ICC. Given its overlapping imaging characteristics with those of hepatocellular carcinoma and other biliary malignancies, an accurate diagnosis often requires comprehensive histopathological evaluation. Molecular analyses, including next-generation sequencing, can further distinguish CLC from conventional ICC because genetic alterations such as Fibroblast growth factor receptor 2 (*FGFR2*), Protein tyrosine phosphatase receptor type T, and AT-rich interactive domain-containing protein 1A (*ARID1A*) mutations are commonly observed.^15)^

Surgical resection remains the primary treatment for CLC with 5-year recurrence-free and overall survival rates of 41% and 72%, respectively.^11)^ However, surgical intervention can be challenging because CLCs often occur in patients with chronic liver disease.^11)^ Regarding chemotherapy, there is no established consensus specific to CLC; however, it is generally managed as a subtype of ICC, with gemcitabine and cisplatin as the standard regimen for unresectable or metastatic disease.^16)^ Additionally, the frequent mutations in *FGFR2* and *ARID1A* suggest their potential role in diagnostic biomarkers and therapeutic targets.^3,15)^ While targeted therapies and immune checkpoint inhibitors have been investigated in intrahepatic cholangiocarcinoma, no clinical studies to date have specifically evaluated their efficacy in cholangiolocarcinoma, likely due to the rarity of this subtype. These findings highlight the need for further research on the molecular landscape of CLCs to facilitate the development of targeted therapies.

Long-term observations of patients with CLCs have provided valuable insights into the clinical behavior of these tumors. CLCs exhibit unique clinicopathological characteristics, including better long-term survival and less invasiveness than conventional ICC.^11)^ A literature review identified five well-documented cases in the English literature with preoperative observation periods exceeding 2 years (**Table 1**). The age of the patients ranged from 15 to 82 years. Initial diagnoses often misclassify the condition as benign entities, such as hemangiomas or dysplastic nodules, highlighting the diagnostic challenges of CLC. Only the present patient was preoperatively diagnosed with CLC. The observation period ranged from 24 to 70 months, with two cases, including the present case, being observed until the tumor more than doubled in size. Notably, three patients showed favorable outcomes despite prolonged observation, in contrast to the typical behavior of ICC.

**Table 1:** Literature review of CLCs in long-term observations over 2 years

Reported year	Age*/Sex	Preoperative diagnosis	Observation period^**^	Surgical procedure	Tumor size^***^	With DPM	Prognosis^**^
2008	76/M	n.d	31	Sub-segmentectomy	10/n.d	n.d	n.d
2012	15/M	Dysplastic nodule	24	Partial resection	11/25	n.d	Alive (30)
2012	69/M	Hemangioma	48	Sub-segmentectomy	12/41	n.d	n.d
2015	41/F	Hemangioma	24	Right hepatectomy	18/15	n.d	Alive (54)
2021	66/M	Hemangioma	60	Left hepatectomy	10/15	n.d	Alive (72)
Our case	82/M	CoCC	70	Right hepatectomy	10/30	Yes	Alive (77)

CLC, Cholangiolocarcinoma.

^*^Numbers indicate years. ^**^The numbers indicated represent months.

^***^The left number is the tumor size (mm) at diagnosis, the right after resection.

This study has some limitations. First, as a case report, it cannot be concluded that the observed clinical course is representative of all patients with CLC. Second, there may have been technical limitations in the biopsy procedures. In the first biopsy, the tumor was small and may not have been accurately targeted. In the second biopsy, the sample may have been taken from the benign-looking component. An earlier diagnosis might have been possible if contrast-enhanced ultrasound had been used to guide the biopsy and sample the early enhancing nodule. However, long-term follow-up, repeated imaging, and pathological evaluations in this case provided valuable insights into the diagnosis and progression of this rare hepatic tumors.

## CONCLUSIONS

This case illustrates the challenges in diagnosing and managing CLC, a rare and under-studied hepatic tumor. Long-term follow-up allows for a better understanding of disease progression and emphasizes the role of imaging and pathology in guiding clinical decision making. Further research is necessary to better understand the biological behavior of CLC and to establish optimal management strategies.

## DECLARATIONS

### Funding

Not applicable.

### Authors’ contributions

YU and GS conceived and designed the study.

TM contributed to data acquisition.

YY contributed to pathological diagnosis and analysis.

GS and MI contributed to the conception and design of the study from the perspective of surgeons.

YY contributed to the conception and design of the study as a pathologist.

YU drafted the paper.

All authors critically reviewed and revised the manuscript for important intellectual content and approved the final version.

### Availability of data and materials

The authors declare that all the data related to this study are available in this manuscript.

### Ethical approval and consent to participate

Ethics approval is not required for case reports, in our institution. Written consent to participate is obtained from the patient.

### Consent for publication

Informed consent for publication of this case report was obtained from the patient.

### Competing interests

The authors have no conflicts of interest to declare and received no financial support for this report.
